# Localization of aggregating proteins in bacteria depends on the rate of addition

**DOI:** 10.3389/fmicb.2014.00418

**Published:** 2014-08-06

**Authors:** Karlton Scheu, Rakinder Gill, Saeed Saberi, Pablo Meyer, Eldon Emberly

**Affiliations:** ^1^Department of Physics, Simon Fraser UniversityBurnaby, BC, Canada; ^2^IBM T.J. Watson Research CenterNew York, NY, USA

**Keywords:** polar localization, protein aggregate, protein induction, nucleoid occlusion, GFP labeled

## Abstract

Many proteins are observed to localize to specific subcellular regions within bacteria. Recent experiments have shown that proteins that have self-interactions that lead them to aggregate tend to localize to the poles. Theoretical modeling of the localization of aggregating protein within bacterial cell geometries shows that aggregates can spontaneously localize to the pole due to nucleoid occlusion. The resulting polar localization, whether it be to a single pole or to both was shown to depend on the rate of protein addition. Motivated by these predictions we selected a set of genes from *Escherichia coli*, whose protein products have been reported to localize when tagged with green fluorescent protein (GFP), and explored the dynamics of their localization. We induced protein expression from each gene at different rates and found that in all cases unipolar patterning is favored at low rates of expression whereas bipolar is favored at higher rates of expression. Our findings are consistent with the predictions of the model, suggesting that localization may be due to aggregation plus nucleoid occlusion. When we expressed GFP by itself under the same conditions, no localization was observed. These experiments highlight the potential importance of protein aggregation, nucleoid occlusion and rate of protein expression in driving polar localization of functional proteins in bacteria.

## INTRODUCTION

Many proteins are observed to localize within bacterial cells. Patterns range from forming ordered groupings of receptors on the cell membrane (Greenfield et al., 2009), to spatial waves (Hu and Lutkenhaus, 1999; Raskin and de Boer, 1999; Loose et al., 2008) to highly localized patterns at either one or both poles (Bowman et al., 2008; Ebersbach et al., 2008; Ramamurthi and Losick, 2009). Such localization has been shown in many cases to play an important role in function, whether in detecting extracellular signals (Alley et al., 1992), to guiding the dynamics of chromosome segregation (Bowman et al., 2008).

**FIGURE 1:**
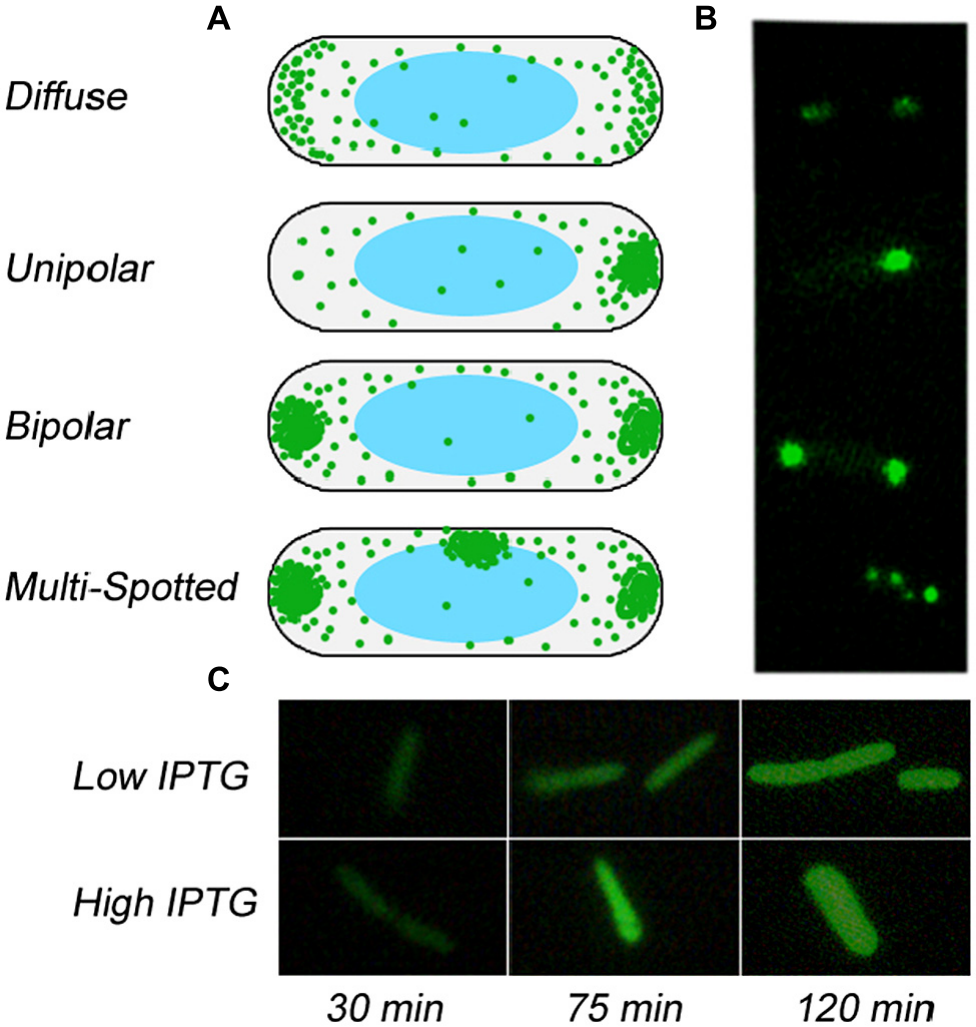
**Possible patterns of localization of aggregates in *Escherichia coli.* (A)** Schematics of the various patterns of aggregating proteins inside a bacterial volume, from diffuse to unipolar to bipolar to multispot. **(B)** Representative images of GFP-tagged protein showing the corresponding localization pattern as depicted in **(A)**. **(C)** Localization of GFP-tagged protein in filamentous cells.

A number of these localization patterns have been shown to arise due to specific interactions of the protein with the bacterial membrane (Alley et al., 1992; Raskin and de Boer, 1999; Ramamurthi and Losick, 2009). Some of these membrane associated proteins are able to localize to regions of specific curvature through lipid mediated interactions (Ramamurthi and Losick, 2009). However some localization has been shown to arise purely within the cytoplasm, without any specific requirements for interactions with the membrane. Indeed some polar localized proteins have been shown to be driven to the poles through their aggregation and occlusion from the central portion of the cell by the nucleoid (Bowman et al., 2008; Ebersbach et al., 2008; Maisonneuve et al., 2008; Winkler et al., 2010). Some of these are functional proteins, such as PopZ that acts as a polar scaffold for many proteins in *Caulobacter crescentus* (Bowman et al., 2008; Ebersbach et al., 2008). But other aggregating proteins, such as those that are misfolded have also shown such localization through the formation of inclusion bodies (Maisonneuve et al., 2008; de Groot et al., 2009; Winkler et al., 2010; Garcia-Fruitos et al., 2011). Recent theoretical (Saberi and Emberly, 2010, 2013; Winkler et al., 2010) and experimental (Bowman et al., 2008; Ebersbach et al., 2008; Winkler et al., 2010; Laloux and Jacobs-Wagner, 2013) work supports the hypothesis that the nucleoid can force aggregating structures within the cell to the poles. Additional levels of control, such as coupling to cell cycle associated spatial oscillations (Laloux and Jacobs-Wagner, 2013) or expression from genes that are spatially localized (Montero Llopis et al., 2010; Kuhlman and Cox, 2012) can further aid the nucleoid driven mechanism. Modeling efforts (Saberi and Emberly, 2013) highlight that the nature of the localization of the aggregate depends strongly on how fast protein is added to the cell, with slow rates leading just to a single polar aggregate whereas faster rates support multiple aggregates at both poles (see **Figure 1**).

A survey of the patterns of all fluorescently tagged enzymes in the *Escherichia coli* genome (Kitagawa et al., 2005) found that a number display either unipolar or bipolar patterning (222 polar localized). In this library (ASKA+), each strain has a high copy number plasmid containing an *E. coli* gene tagged with green fluorescent protein (GFP) whose expression is controlled by the *lac* promoter. The reported patterns were generated by expressing each strain with the same level of inducer, in this case IPTG. Could the polar localization of these proteins be the result of sequence specific localization cues, or potentially due to aggregation and nucleoid occlusion as well? The latter is predicted to lead to a strong rate of expression dependence on the resulting polar pattern. To explore whether aggregation plus nucleoid occlusion could be the mechanism behind a portion of the polar localization observed in *E. coli*, we tested whether we could alter the observed patterns in a manner consistent with the predictions of theory by simply changing the rate of expression of protein (Saberi and Emberly, 2013). To do this we started by selecting five, otherwise arbitrary, globular proteins that have no transmembrane domain and that showed some form of polar localization in the initial screen of the ASKA+ library. For each strain we then imaged the formation of localization patterns of the GFP-tagged protein in time at various levels of induction. The model predicts that the localization should depend strongly on how fast the protein is added. If, however, localization is specific to particular cellular locations due to sequence, then the rate of addition should not affect localization significantly; the localization should appear once the concentration crosses some threshold for the specific binding, which does not depend on rate. From our experimental analysis, we find that for each strain tested, the observed localization is indeed rate dependent that is at least consistent with the predictions of the model based on aggregation with nucleoid occlusion.

## MATERIALS AND METHODS

### SAMPLE PREPARATION

Five samples of *E. coli* K-12, strain AG1 [recA1 endA1 gyrA96 thi-1 hsdR17 (rK- mK+) supE44 relA1], that have different plasmids inserted in them were obtained from the NBRP-*E. coli* at NIG ASKA+ Library (Kitagawa et al., 2005). The plasmids contained a lac promoter and one of the genes found in *E. coli* tagged with GFP (λem: 509 nm, λex: 395 nm). The different *E. coli* cells will be referred to by the name of the gene on their plasmid (*glnQ, pykA, cysJ, aceA,* and *pfkA*). Cells were grown at 37°C for 17 h in Luria-Bertani (LB) medium [1% Bio Tryptone (Bioshop), 0.5% yeast extract (EMD), 1% NaCl] containing 14 μg/ml Chloramphenicol (BDH Chemicals) for selection. The cells were then diluted 1:50 and grown for 3 h to be well into log phase. Isopropyl β D thiogalactoside (IPTG) was then used in varying concentrations (0.025, 0.05, 0.075, 0.1, and 0.25 mM) to induce expression of the cloned gene.

### FLUORESCENT MICROSCOPY AND IMAGE ANALYSIS

After induction, bright field and fluorescent microscopy were performed (Zeiss Axioskop with Photometrics CF) for 2 h in 15 min intervals. About 60 cells were photographed at each time step. Afterward the GFP intensities of imaged cells were quantified and the resulting pattern was classified by eye as either non-fluorescing, diffuse, unipolar, bipolar, or multi-spotted (see **Figure 1B** and Figures S1–S3). Bipolar patterns clearly show a circular GFP spot at each pole (see Figure S1B), whereas at low expression rates some diffuse patterns show some increased crescent shaped GFP fluorescence at both poles that is easily distinguishable from a bipolar pattern based on both spatial and significant intensity differences (see Figure S1B and Figure S2). Filamentous *E. coli* cells were not included in the count. Cells that appeared in the fluorescent image but not the bright field image were also not included in the classification of patterning. At each time point the fraction of each localization pattern within the total number of characterized cells was evaluated.

## RESULTS

Our model for polar localization argues that protein aggregation along with nucleoid occlusion can cause an aggregate to form at one or both poles depending on how fast the aggregating protein is added to the cell (Saberi and Emberly, 2013). Motivated by this model, we explored whether it may be at work in the patterning of polar localizing tagged proteins in the ASKA+ *E. coli* library (Kitagawa et al., 2005). We selected five genes (*glnQ, pykA, cysJ, aceA,* and *pfkA*) from this library whose proteins showed polar localization to see if we could alter their localization patterns by changing the rate of addition. The proteins were selected because they were globular, had no transmembrane domain and showed polar localization in the reported images in the initial screen of the ASKA+ library.

Since each strain could potentially possess different numbers of plasmids and therefore express protein at different rates under otherwise identical conditions, we chose to grow up each strain at several induction levels. (In Figure S3 we show the increase of GFP intensity with time at different levels of induction, showing how higher amounts of IPTG lead to higher expression rates). This would allow us to make correspondences between the strains in terms of expression rates. Each strain was grown up on six different levels of IPTG (0.025, 0.05, 0.075, 0.1, and 0.25 mM) leading to a different rates of expression for each GFP tagged protein. The cells were then imaged every 15 min, with the patterns of each imaged cell recorded into one of four patterns (see Figures S1, S2): diffuse, unipolar, bipolar, and multi-spot (see **Figure 1B**, Figure S1A). For each strain, GFP starts to come on 20 min after induction, and so we have very few fluorescing cells at the earliest time points, making it a challenge to fully characterize the nature of the pattern at times less than 30 min. Only one of the five strains, *cysJ*, showed considerably lower levels of GFP induction, and hence less cells to characterize for patterning and was left out of the analysis that follows.

**FIGURE 2:**
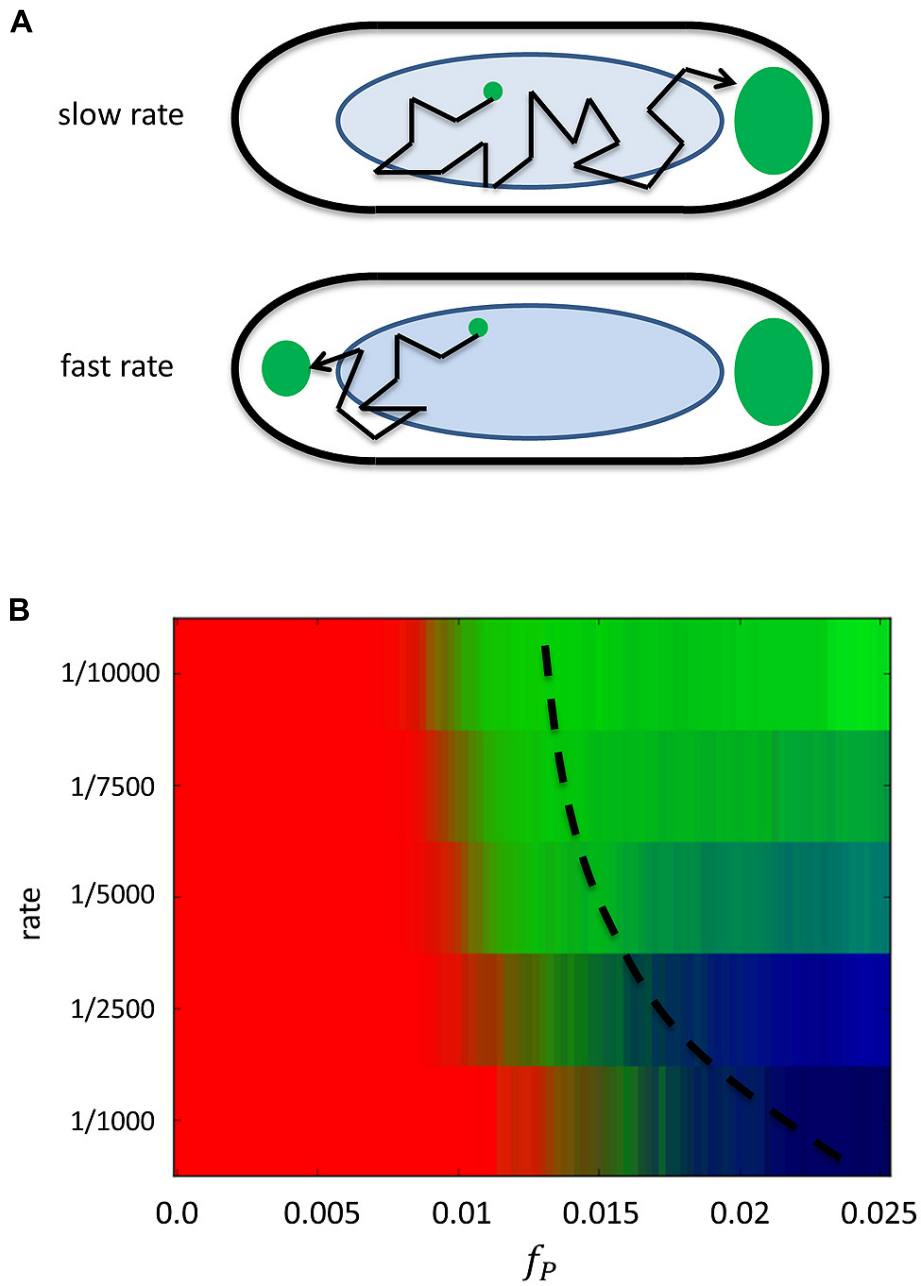
**Predicted dependence of localization on the addition rate of protein. (A)** Schematic of the model for polar localization. Top cell has a slow rate of protein addition where the existing aggregate will tend to capture all added proteins. The bottom cell has a fast rate of addition, so that not all proteins will be captured, leading to the possible formation of another polar aggregate. **(B)** The calculated probability of observing a particular localization pattern as a function of the amount of protein f_P_, in the cell at different rates of addition. Each row corresponds to a given rate of addition and protein is added to the cell leading to a monotonically increasing amount with time (*x*-axis).Thus each row represents the temporal evolution of the localization pattern as protein is added at the given rate up to some final amount. At slow rates of addition (rate <1/7500), the pattern transitions from diffuse (red) at low amounts of protein to unipolar (green) at higher amounts. At faster rates of addition (rate >1/2500), the pattern transitions from diffuse to bipolar (blue) at higher concentrations. Also shown is a hypothetical dashed line for the final value of f_P_, if protein is only added for the same amount of time at each rate.

The model predicts that at slow rates of addition, only one polar aggregate should form, since all added protein will have time to diffuse and eventually get captured by the growing aggregate. This is shown in **Figure 2B**, which plots how the pattern changes as protein is added to the cell in time at a given rate of expression. At low rates, as time progresses and the amount of protein f_P_, increases in the cell, the pattern transitions from being predominantly diffuse (red) to unipolar (green). At higher rates of expression, as more and more protein is added to the cell the pattern goes from being diffuse to a brief period of being unipolar and finally to bipolar (dark blue). The localization behavior at both low and high rates of expression can be understood in terms of a diffusion-to-capture model that has been used to explain receptor localization on the bacterial membrane (Wang et al., 2008; Greenfield et al., 2009), except here in the context of the cytoplasm (see **Figure 2A**). At low rates of addition added proteins have enough time to be captured by a lone aggregate before the next protein is added, causing it to continue to grow. At faster rates, diffusing proteins do not have time to cover the full length of the cell and eventually reach densities that are sufficient to start a new aggregate. It is entropically and energetically favorable for aggregates to migrate to the poles due to the entropic force exerted on them by the presence of the bacterial nucleoid and the ability to grow to larger sizes in the polar regions where there is less DNA.

For each strain we could find a concentration of IPTG that lead to a rate of expression of the GFP tagged protein such that unipolar patterning dominates the population at later times (see Figure S4). At early time points the pattern is diffuse and transitions to being predominantly unipolar ~80% at the final time point. At these low induction concentrations, some bipolar patterning exists, but at very low levels in all strains. For the strain expressing CysJ, similar behavior was observed, though the statistics are based on only a few fluorescing cells (data not shown).

As the rate of addition of protein is increased by increasing the concentration of IPTG, the model predicts that patterning should move from being predominantly unipolar to that of bipolar (or possibly multi-spot) as (see **Figure 2B** at the higher rates). This should occur at a rate that goes as r ~ D/L^2^ where *D* is the diffusion coefficient of the soluble protein and *L* is the length of the bacterial cell. [For GFP in *E. coli*, *D* = 7.7 μm^2^/s (Elowitz et al., 1999) and *L* = 2–3 μm, so *r* = 0.9–2 GFP/s as an upper estimate]. At rates faster than this, the pattern transitions from unipolar to bipolar since another aggregate can form in the cell, moving to the other pole. To test whether this would happen in the chosen set of GFP-tagged proteins, we grew up each strain in increasingly higher levels of IPTG. For each strain we found that at a particular higher level of IPTG, and hence a faster rate of addition (see Figure S3), bipolar patterning dominates at longer times (see Figure S5). Thus adding protein at a faster rate leads to the formation of another aggregate that is then occluded by the nucleoid to the other pole.

**FIGURE 3:**
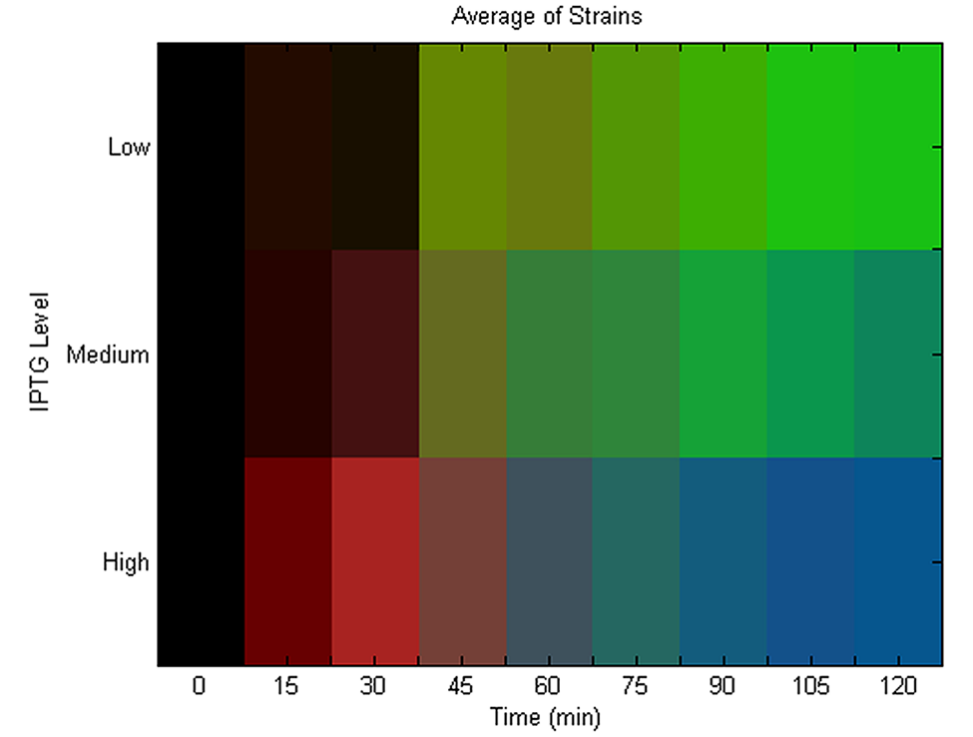
**Average localization pattern versus time and induction rate.** Population and strain averaged localization pattern versus time at different induction levels. The RGB color represents the mix of patterns at each time point from diffuse (red), unipolar (green) to bipolar (blue). For each strain, an induction level was found that lead to unipolar patterning at late times (“Low”; see Figure S4) and also bipolar (“High”; see Figure S5). The IPTG concentration in between the “Low” and “High” concentration for each strain was selected for the midpoint expression level (“Medium”). The average localization pattern over all strains was then calculated for each of these induction levels. The population average for diffuse (red), unipolar (green) and bipolar (blue) is then plotted as an RGB level at each time point to make the heat map, and where black represents time points that had no data.

We summarize our experimental findings in **Figure 3** that shows the average localization pattern over the population for all strains at different IPTG levels versus time. This shows that the localization behavior of these GFP tagged polar localizing proteins depends on the rate of addition. This finding is consistent with the predictions of the model (see **Figure 2B**), namely that unipolar patterning dominates at slow rates of addition, transitioning between a mix of unipolar and bipolar at intermediate rates to finally bipolar at the highest levels of induction. We note that we do not detect much of a diffuse phase at early time points at low rates of expression. As we previously mentioned, characterization of the pattern at early time points is compromised due to the slow folding time of GFP. **Figure 3** is also an average over all strains, and looking at each strain individually (Figure S4), pykA shows the diffuse pattern being present at early time, and similarly for the other strains if the diffuse data is extrapolated to earlier times. As a control, we expressed GFP alone under the same conditions – here at 0.025 and 0.25 mM (see **Figure 1C**). Under both levels of expression, no localization could be detected. Thus for the five strains tested there is some form of specific interaction that exists between the tagged proteins leading to their aggregation that is either inherent to the native protein or arises due to the tagging. This aggregation in the presence of the bacterial nucleoid leads to their localization to the poles.

## DISCUSSION

Protein aggregation leading to polar localization within the cytoplasm of bacteria has many functional consequences, from regulating signaling, to tethering chromosomes, to segregating misfolded proteins to guard against potential deleterious consequences. Such localization could be targeted by spatially specific cues (Montero Llopis et al., 2010; Kuhlman and Cox, 2012) or aided by actively driven processes as recently shown for the polar localized scaffold protein PopZ (Laloux and Jacobs-Wagner, 2013). Modeling efforts have shown that nucleoid occlusion in addition to protein aggregation can be a sufficient mechanism to drive the spontaneous formation of polar localization.Our theoretical modeling has shown that such polar should have strong dependence on the rate at which protein is added to the cell. To explore this prediction we selected five cytoplasmic proteins that showed polar localization when tagged with GFP, and expressed them at different rates within the cell. The mRNA is expressed off of a high-copy number plasmid and so the resulting GFP-tagged proteins should be produced uniformly within the cell. In all cases we found that there was a strong dependency on the rate of protein addition consistent with the predictions of the model of nucleoid occlusion plus protein aggregation.

It is possible that the interactions leading these polar localized proteins to aggregate is native to these proteins and is essential for them to target either one or both poles. Indeed, as our experiments show, the selection of unipolar versus bipolar localization can be selected for by tuning the rate at which the protein is expressed in the cell. In principle the bacteria could tune such expression levels to select for particular polar patterns. One prediction is that one might see rate dependent polar patterning for constitutively expressed genes in bacteria that functionally aggregate. Examining the localization data obtained in the recent library of chromosomal YFP tagged genes in *E. coli* would give a potential test of this prediction (Taniguchi et al., 2010). Another possibility that could explain some of the polar localization is that it results due to tagging with GFP as recently highlighted (Landgraf et al., 2012). The tagging by GFP for certain proteins leads to their aggregation and formation of inclusion bodies that then can be driven to the poles via the mechanisms put forward here. Our own findings for expressing GFP alone showed no preference for localizing to the poles. Nevertheless, further work using antibody staining or other fluorescent tags would help to further clarify whether the localization is the wild-type pattern or not. It should also be noted that the theoretical modeling revealed that initial conditions play a strong influence on the dynamics of the resulting pattern. Had there been a pre-existing pattern present due to the wild-type protein, once the tagged protein is introduced it would then get quickly incorporated to what is already there. Our experimental findings show an emergence of localization patterns for the GFP tagged protein arguing against there be any strong initial conditions. Categorization and functional testing of these polar localized proteins will give further clues into the biological significance of using nucleoid occlusion and protein aggregation as a method for localization.

## Conflict of Interest Statement

The authors declare that the research was conducted in the absence of any commercial or financial relationships that could be construed as a potential conflict of interest.
