# Supplementary material for: Localization of aggregating proteins in bacteria depends on the rate of addition
Source: Front Microbiol. 2014 Aug 6;5:418. doi: 10.3389/fmicb.2014.00418 (PMC4123723; doi:10.3389/fmicb.2014.00418)
Supplement: Supplementary file 1 [file Presentation_1.ZIP › Supp Mat legends.pdf]

Figure S1: Observed patterns across all strains. (A) Representative images of diffuse, unipolar and bipolar patterns in each of the five strains. (B) A lineplot of GFP intensity for the images shown for pykA in (A), showing the clear distinction in intensity between diffuse and patterns that possess polar localization.

Figure S2: GFP intensities for the different patterns. Distribution of integrated GFP intensities for diffuse, unipolar and bipolar classified cells across all strains.

Figure S3: IPTG dependent protein expression rate. Population average integrated intensity versus time at different rates of induction for the pykA strain. The IPTG amount in milimolar is shown in the legend. The typical number of cells identified from DIC images at each time point was n=55.

Figure S4: Observed localization patterns at low rates of addition in ASKA+ clones. Fraction of each localization pattern for GFP expressing in each strain as a function of time at low levels of IPTG. At these low rates of expression, all strains show unipolar patterning (red) as the dominant form of localization. At each time point around n=60 cells were measured. Given the number of cells evaluated the statistical error is estimated to be ~ 12 - 20 % at each time point for the various patterns. These same statistical uncertainties apply to Fig. S5.

Figure S5: Observed localization patterns at fast rates of addition in ASKA+ clones. Fraction of each localization pattern for GFP expressing in each strain as a function of time at higher levels of IPTG. At these higher rates of expression, all strains show bipolar patterning (blue) as the eventual dominant form of localization.
