# Supplementary figures and images for: Localization of aggregating proteins in bacteria depends on the rate of addition
Source: Front Microbiol. 2014 Aug 6;5:418. doi: 10.3389/fmicb.2014.00418 (PMC4123723; doi:10.3389/fmicb.2014.00418)

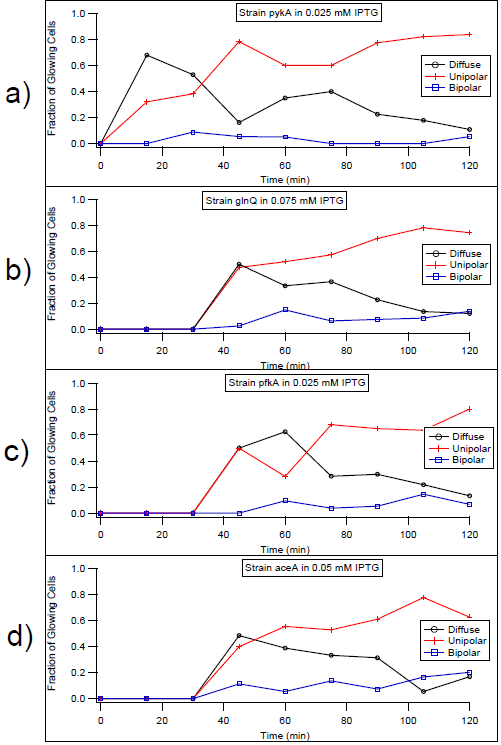

Supplement: Supplementary file 1 [file Presentation_1.ZIP › Supp Fig 4.TIF]

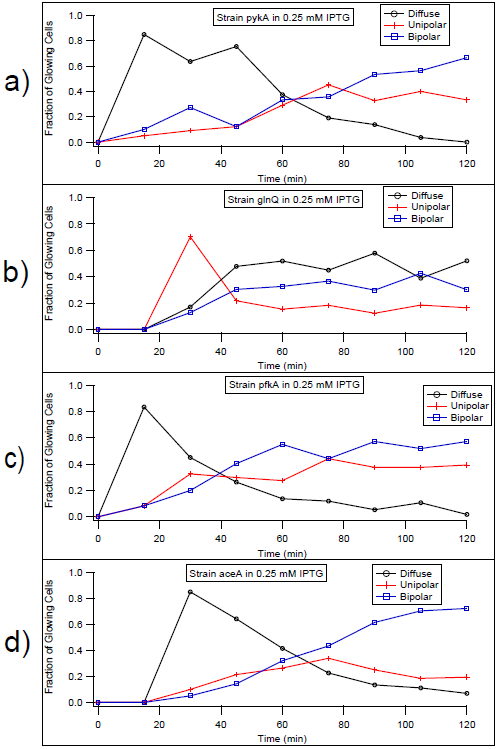

Supplement: Supplementary file 1 [file Presentation_1.ZIP › Supp Fig 5.TIF]

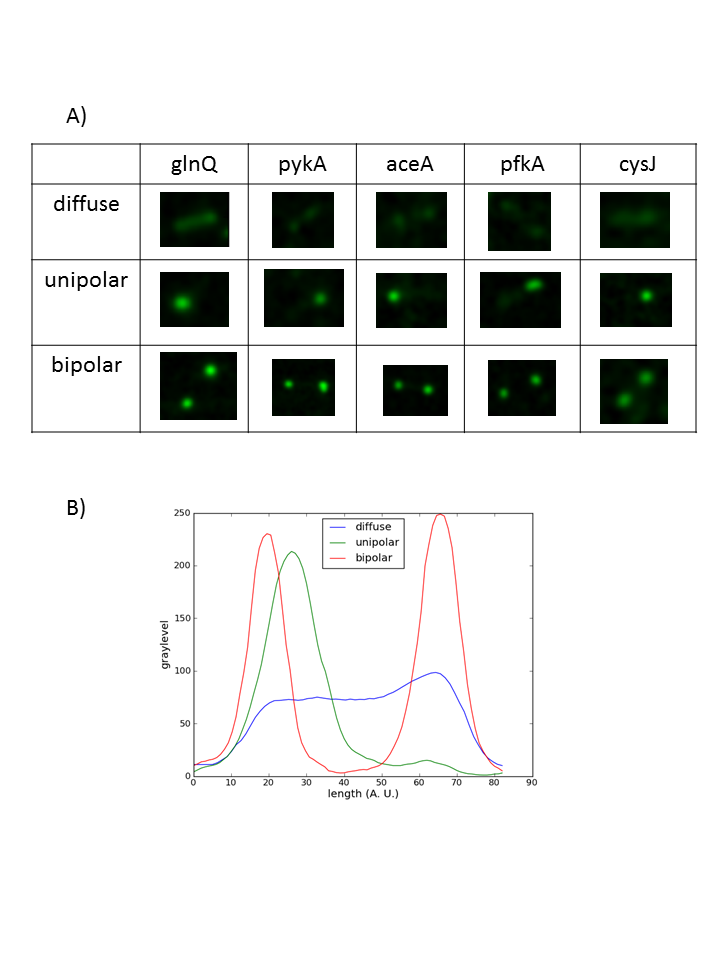

Supplement: Supplementary file 1 [file Presentation_1.ZIP › Supp Fig 1.TIF]

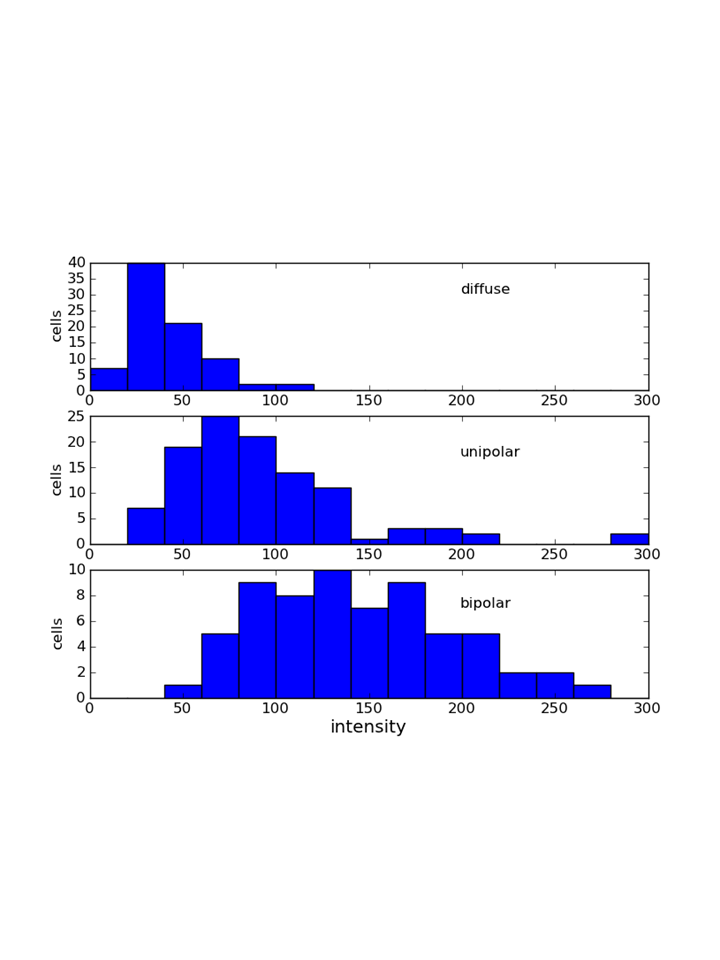

Supplement: Supplementary file 1 [file Presentation_1.ZIP › Supp Fig 2.TIF]

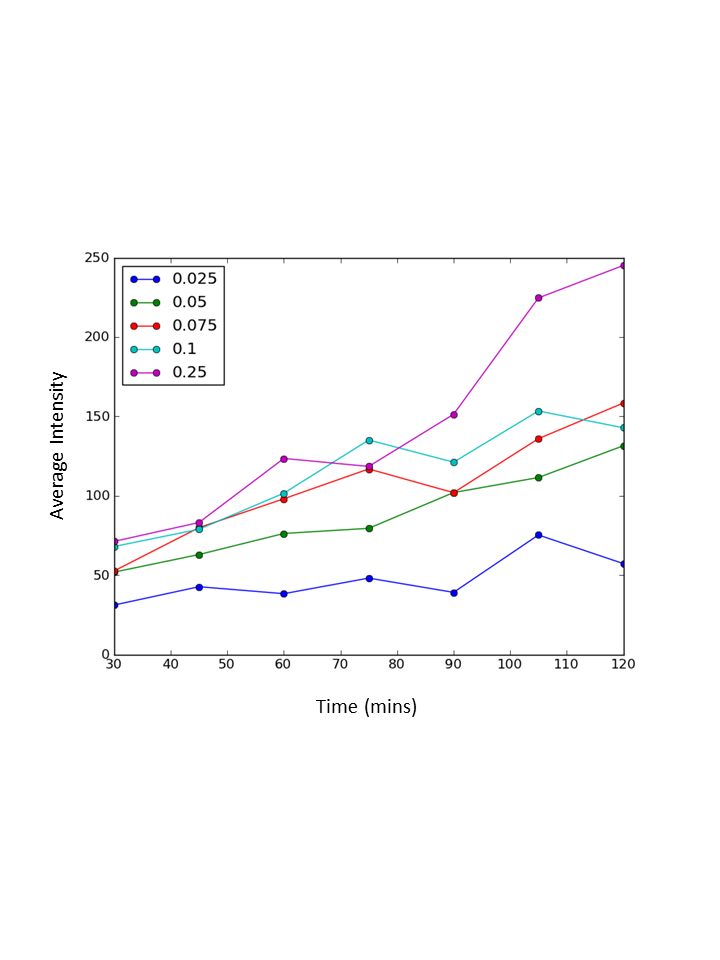

Supplement: Supplementary file 1 [file Presentation_1.ZIP › Supp Fig 3.TIF]
